# Dental Legislation

**Published:** 1903-03-15

**Authors:** James A. McGeough

**Affiliations:** Boston


### DENTAL LEGISLATION.

BY JAMES A. MC GEOUGH, OF BOSTON.

Being an Extract of an Address Delivered before the Massachusetts Dental Society.

Why should there be any law in relation to the practice of dentistry? A glance at the history of the profession—what it is and what it has been—will help to explain. The contrast is interesting and instructive.

Formerly, and not so very long ago either, the practice of dentistry was anything but attractive. If dentistry was known and practiced among the ancient Egyptians in any degree of perfection, as is claimed by some, it certainly became one of the lost arts. As now practiced, I understand dentistry is of a very modern date—fifty years ago; and perhaps some of the older members of the profession to-day may fairly claim to have been at its birth. Prior to that time and for the centuries before, there was little need of any law for the regulation of the practice of dentistry, although they do tell some harrowing tales of the practice in those early days.

If the doctors ever performed any dental operation at all, it was only in case of dire necessity, and the less they had of the business no doubt the better they liked it. In those olden times the practice consisted chiefly in the extracting of fractious and troublesome teeth, and we are told it was main strength on the one side and heoric endurance on the other, as the operation was performed with instruments something like a blacksmith's forceps and a cold chisel. In those happy days it must have been easy for a dentist to collect his bills, for after being dragged about the floor and generally used up, as the stories have it, the patient must have considered that the operator had earned his fee. But sometimes, it is said, things took a different turn. When the suffering patient happened to be a big man with a bad toothache and a worse temper, the operation sometimes assumed the character and proportions of an incipient prize-fight, and as between patient and practioner it was often a question as to which was the better man.

In those pre-historic days of the profession there was little or no competition. A dentist's office was hard to find. A suffering patient had no difficulty in making a date if he found his dentist; at least there was no crowding or waiting for turns in those days, and seldom, if ever, was there any occasion to invoke the law. Now all this is changed. Dentistry to-day is in comparison a pleasure and a pastime. A dental office is no longer a place of torture, put a "parlor" of luxury and ease, and the throng of patients from day to day attest the popularity and success of the profession. The reason of all this is obvious. We may read it in the signs of the times. Daily from my office window, in great luminous letters of heroic size, so that all who run may read, and a long way off too, if necessary, I am compelled to notice these fascinating specimens: "Big Dental Office," "Teeth extracted without Pain," "Lowest Prices in Boston for Artificial Teeth and all Dental Work," with attractive pictures of the work and specimens of the teeth extracted. What the size of the office has to do with the practice in these days, I don't know. It may be to accomodate the waiting and anxious public. Then, as a part of this same sign, in smaller, but still attractive, letters, I read, "Examinations free," "Bridge-work, $5.00," "Gold crown, $5.00," "Headquarters for artificial teeth, full set, $5.00." On a building front in the next block there used to be a sign, "Full set, $2.50," but that has been recently taken down. I suppose the lucky dentist got rich and retired. But the most interesting of all these alluring signs is this, in small confidential type: "Dr. ——— the *real* painless dentist." It is so comforting to know that while you are being painlessly operated on, the operator himself is also free from pain. These, I understand, are but samples of what we now find in many parts of the city of Boston, and in every other city and town of importance throughout the country. Even in the street-cars these dental signs now occasionally greet us, and regularly in the daily papers they may be seen in gay and festive columns with "Red Fox Ale," "Divorces procured on the installment plan" "Clothing on credit; no money down," "Consumption treated, no cure no pay," and cheerful announcements of that kind, with others too numerous and some too unedifying to mention. But I am not discussing the propriety or impropriety of public advertising. I am simply stating some familiar facts, to show the wide-spread and growing popularity of the science itself as affecting the rights and interests of the people.

Then again, dentistry as practiced to-day, with all its improved and painless methods, is not free from danger. Grave and serious operations come at times to the dentist's office, and even in minor operations, with the use of anesthetics, death in the dental chair is not unknown to the profession.

From this condition of things which I have thus endeavored to outline, the reason and necessity for a law regulating the practice of dentistry must be apparent. But to be a little more explicit, it is important to observe in this connection that all good law is and must be for the benefit of the many, not the few, It is a mistake to suppose, as we sometimes hear, that the law is for the benefit of any particular number or class of persons. There should be no favoritism under the law. "The greatest good of the greatest number" is the object in view. Incidentally, the individual may and often does reap some benefit or reward, while, on the other hand, some may suffer more or less privation, but the true and only legitimate purpose and object of all law is the welfare and protection of the public. *Salus populi suprema est lex.* This is the test. If it meets this requirement without imposing any unreasonable hardship anywhere, it is a good law, and that this is the true rule a moment's reflection will show.

In primitive society and isolated settlements there is little need of any law. Robinson Crusoe and his man

Friday on their lonely island didn't need any law, and in a small Christian community like Arcadia, for instance, about which our own Longfellow so beautifully wrote, the ten commandments no doubt would be sufficient. But in the great cities and centers of population an altogether different condition of things exists. The people outside of little circles, more or less extended, although forming the same general community, are practically strangers and unknown to each other, to say nothing of the great floating population in every large city who can have little or no acquaintance of place or people, and yet all these are entitled to and need the protection of the law. Then the difficulties and necessities of life in these days are increased a thousand-fold. The competition in business and trades of all kinds has become very great. There never seems to be money enough to go round, and in the race of life, or rather in the struggle for very existence, the great majority are constantly in danger of being borne to the ground in every direction, and hence the necessity for more specific laws and regulations for their protection and relief.

Now, this rivalry, competition, and strife we find not only in all trades and branches of business, but in the professions as well, and dentistry is no exception to the rule. Incited by the growing commercialism of the times, and attracted by the opportunities which this comparatively new field for money-making affords, it is not to be wondered at if many seek the profession for the money there is in it, while some of those already in, not members of this society of course, are tempted, I fear, to adopt methods and practices in which the good of the public or honor of the profession has little to do. Accordingly, in Massachusetts, and I understand in most of the States of the Union, there are laws for the regulation of the practice of dentistry.

When you have some plumbing to be done at your house, you want to be assured that the health of your family is not endangered by the faulty construction of an incompetent workman. If you have a prescription to be filled, you want to feel that you may enter any drug-store and have it filled without the fear of being poisoned by the wrong medicine. If a lawyer is to be employed, it is important that he should be a member of the bar. If a doctor or a dentist is wanted, it is only fair to the public that the individual called upon is competent to perform the services required. This is the object of the law, and this the law endeavors to secure.

Now, in the practice of dentistry, as in the practice of any other profession, trade, or calling in which the safety of the public is concerned, there are two ways, and, when you think of it, only two ways, in which the rights and interests of the public are or can be reasonably cared for: First, by admitting to practice those only who are qualified, and secondly, by holding those who are admitted to an exercise of professional skill, due care, and dilligence, and to meet these requirements we have two kinds of law—the statute or written law, and what we call the common or unwritten law.

Let me speak of this second kind of law first, because it is the earlier of the two and in a practical sense perhaps of less importance. I trust I will not offend the strong New England Americanism of any one present when I say that this common law we get not from any legislation of our own State or National, but we get it, as we get our language, from what may be very properly called in a limited sense the mother-country—England. It came over in the "Mayflower." It was the guide of the thirteen original colonies before the revolution, and to-day it is that great body of jurisprudence, modified and changed, by statute more or less to suit the varying conditions of time and place, but still the great body of jurisprudence under which we live. Broad and comprehensive in its scope, it governs and regulates the people in all their rights, duties, and obligations among and to each other. that of master and servant, employer and employed; doctor and patient, lawyer and client. It includes all those fundamental rules and principles of right and justice recognizad in and binding upon all our courts, civil and criminal, and it has been adopted by every State in the Union, except Louisiana alone; and this law we get from England.

Under the provisions of this law every practitioner is bound to a reasonable degree of professional skill and the exercise of of due care and diligence, and if by reason of any lack of this professional skill or diligence a patient sustains an injury at his hands, he thereby becomes liable to a suit for damages under the common law.

The practitioner, who through a want of professional skill or due care injures a patient is liable therefor under the common law in a suit for damages. This is now the law, always has been the law in Massachusetts, and, I venture to add, always will be, because founded on the unalterable decrees and principles of justice. But upon reflection, it will be easily seen that this did not meet the entire difficulty. A right of action was all very well, but few cared to adopt the remedy. The expense of litigation, the proverbial law's delay, the desire to avoid publicity, and very often the unaccountable indifference of individuals, especially in large communities, as to their own rights left the people an easy prey to every advertising, unscroupulus mountebank who might chose to enter and disgrace the profession. Anybody and everybody, with or without a college training or certificate of qualification, had a right to practice. Every college and dental school throughout the country, reputable and disreputable, had its own standard of qualification, or want of standard, and as to who were competent or incompetent the indiscriminating public had no means of knowing. Hence, the reason and necessity, for further legislation. And to meet this condition of things the statute of 1887 was passed by our legislature. This statute, with few subsequent amendments, is now embodied in Chapter 76, Sections 24 to 29 inclusive, of the Revised Laws, which came into effect January 1st, and have been lately published. With the provisions of this law the society and members of the profession are of course familiar, and it needs no defence.

Every law to be just must be impartial, reasonable in its requirements, and for the good of the public. And this fairly describes our Massachusetts dental law. The controlling feature is that only those who show themselves to be duly qualified by a satisfactory examination before the State Board of Registration shall be permitted to practice. All else is detail.

The advantages of this law must be apparent. Chiefly, it establishes a central board of examiners who are appointed by the governor and directly responsible to the State; it fixes a high and a uniform standard of qualification throughout the State which no other system can provide, and if properly and judiciously enforced it secures to the public that reasonable measure of protection which it is the duty of the Commonwealth to furnish. Besides, the moral effect of such a law is not without its influence. It maintains the high standard of the profession and inspires the confidence of the public. The honest practitioner gives it his hearty approval and support. The laggard and the charlatan alone complain.

There is one other point to which I wish to call attention, and I have done. The vindication of very good law lies in its prosecution. Useless and in vain is that law which may be violated with impunity. A law that becomes a dead letter is worse than no law at all. Every law to be effective must be enforced. These are acknowledged truisms. Now under the common law, as we have seen, those only who suffer injury are entitled to sue for damages, so that the the prosecution of the common law in thls particular must be left to them. Prosecutions under this Statute Law belong on the criminal side of the court and principally devolve upon the State. The Board of Registration, among other things, is charged with the important duty of regular examinations and the issuing of certificates to successful applicants, but as to who shall undertake the duty of prosecuting violations of the law the statute itself is silent, and accordingly this duty rests chiefly with the regular police authorities. Of course, the board can not be indifferent to open and flagrant violations of the law, and in my opinion it is entirely competent for them. as a board or as individuals to furnish the police authorities with such facts and evidence as may come to their knowledge. But this criminal prosecution is at best an unpleasant duty, and as the law makes no provision for the necessary expense and loss of time this work involves, farther than as I have indicated the board can scarcely be expected to go.

It is also entirely proper and competent, in my opinion, for this society, as a whole, or by its duly appointed committees in the various cities and towns to take such action and give such assistance to the local police in this respect as the society may from time to time think best and determine.

But after all is said and done under the law, much must be left to the individuals conscience, the good influence of the society, and public opinoin. The charlatan who contrives to get into the fold and the reckless practitioner, however incompetent, who only thinks of the money there is in it, and just escapes the law, must eventually find their proper level and be relegated to that limbo of unenviable practice and standing in the community which their effrontery, practice and their conduct so richly deserve.

Money is not all there is to live for. Good name in every honorable profession at least should count for something, and in this connection I would recommend for the serious consideration of the society an amendment to the present statute law, to the effect that the conviction of any practioner of a felony or lesser crime involving the breach of any professional trust or serious malpractice shall operate as a cancellation of his certificate and a bar to future practice. The profession can ill afford to lower or permit to be lowered that degree of professional skill, conscientious service and high moral standing which the good name and honor of the profession itself require and the general public, upon which it depends, have a right to demand.—*International.*

